# Differential expression of microRNA between normally developed and underdeveloped female worms of *Schistosoma japonicum*

**DOI:** 10.1186/s13567-020-00851-4

**Published:** 2020-09-25

**Authors:** Yu Han, Jintao Feng, Yuqi Ren, Luobin Wu, Hao Li, Jinming Liu, Yamei Jin

**Affiliations:** 1 Shanghai Veterinary Research Institute, Chinese Academy of Agricultural Sciences, Key Laboratory of Animal Parasitology, Ministry of Agriculture, No.518, Ziyue Road, Minhang District, Shanghai, 200241 PR China; 2 College of Life Sciences, Shanghai Normal University, Shanghai, China

**Keywords:** *Schistosoma japonicum*, microRNA, deep-sequencing, differential expression, predicted target genes

## Abstract

Eggs produced by bisexual infected mature female worms (MF) of *Schistosoma japonicum* are important in the transmission of the parasite and responsible for the pathogenesis of schistosomiasis. The single-sex infected female worms (SF) cannot mature and do not produce normal eggs; also they do not induce severe damage to the host. In this study, the microRNA (miRNA) expression profiles of 25d MF and 25d SF were investigated through Solexa deep-sequencing technology to explore the developmental mechanisms of schistosome female worms. There were 36 differentially expressed miRNA, 20 up-regulated and 16 down-regulated found in MF/SF worms, including some development related miRNA such as bantam (ban), let-7, miR-124, miR-8, miR-1, miR-7. There were 166 target genes of up-regulated miRNA and 201 target genes of down-regulated miRNA after comparing the target gene prediction software results with RNA-Seq transcriptome results. Analysis of the target genes shows that different ones are involved in MF and SF worms in Gene Ontology terms, with a similar situation in KEGG. This observation indicates that different genes regulated by differentially expressed miRNA take part in MF and SF and lead to differential sexual status. This means that the sexual status of female worms is regulated by miRNA.

## Introduction

Schistosomiasis is an important helminth infection that mainly occurs in developing countries. It is endemic in 74 countries, with roughly 120 million individuals being severely affected [1]. No successful vaccine is currently available for this disease. Praziquantel is the only and most extensively used drug for treatment, but it is ineffective against young worms [2] and there are concerns that drug resistance to praziquantel may be developing [3]. Consequently, there is an urgent need to identify novel drug targets and/or develop alternative therapeutic strategies.

In schistosomiasis, the severely pathological injuries to the final host are caused by the amounts of eggs produced by mature normally developed female worms (MF). These eggs are also responsible for the spread of the disease [4], but the underdeveloped female worms (SF) which come from single-sex infection cannot develop normally or produce normal eggs. So these single-sex infected worms (female or male) will not seriously damage the host, and will not induce the prevalence of schistosomiasis. Based on the important role of eggs produced by mature females, the developmental mechanisms of female worms is the research target for many schistosome researchers.

MicroRNA (miRNA) are a class of small non-coding RNA (18–25 nucleotides in length) that are generated from endogenous transcripts [5]. They function as guide molecules for post-transcriptional gene regulation by base-pairing to their messenger RNA (mRNA) targets [6], they bind to the target and induce mRNA translation repression and/or exonucleolytic decay. Through these actions, miRNA regulate gene expression during development, differentiation, proliferation, death, and metabolism in many organisms [7]. To date, several groups have carried out studies to identify miRNA in schistosomes to provide some insight into the role of miRNA in schistosome development [8–10].

In this study, the expression profiles of miRNA of 25d MF and SF *Schistosoma japonicum* (*S. japonicum*) were investigated. Based on the analysis of the differentially expressed miRNA and their predicted target genes, the correlation between miRNA and the mature status of female worms was studied.

## Materials and methods

### Ethics statement

All animal experiments were conducted in accordance with the guidelines of the Committee for the Care and Use of Laboratory Animals of Shanghai Veterinary Research Institute, Chinese Academy of Agricultural Sciences (permit no. SYXK-2016-0010). The study protocol was approved by the Ethics and Animal Welfare Committee of the Shanghai Veterinary Research Institute, Chinese Academy of Agricultural Sciences.

### Host animals and parasites

BALB/C male mice, 4- to 6-week-old, were used for worm collection. *S. japonicum* (Chinese strain) worms were maintained in mice and *Oncomelania hupensis hupensis* snails at the Shanghai Veterinary Research Institute, Chinese Academy of Agricultural Sciences (SHVRI, CAAS). Cercariae of *S. japonicum* were shed from infected snails after exposure to artificial light for 5 h at 25–28 °C. The 25d MF were collected from bi-sexual infected mice at 25 days post-infection by perfusion. Worms were washed five times in phosphate buffered saline (PBS, pH 7.4) at 4 °C to remove excreted and secreted proteins and other interfering substances and were then incubated at 4 °C for 30 min. The paired worms spontaneously separated into females and males, then the female worms were collected. The 25d SF were obtained after perfusion from mice infected by female cercariae. The sexes of cercariae shed from individual snails were identified by polymerase chain reaction (PCR) based on the patent: CN 101597645. Then the female worms were washed five times in PBS at 4 °C to remove excreted and secreted proteins and other interfering substances, then collected.

### RNA extraction, small RNA library construction and sequencing

Total RNA was extracted using TRIzol reagent (Invitrogen Life Technologies, Carlsbad, CA, USA) according to the manufacturer’s instructions. The quality of RNA was measured using a NanoDrop 1000 spectrophotometer (Thermo Fisher Scientific, Waltham, MA, USA). The RNA samples were stored at -80 °C.

The construction of small RNA libraries was carried out using the TruSeq Small RNA Sample Prep Kit (Illumina, San Diego, CA, USA) according to the manufacturer’s protocol. Briefly, total RNA was subjected to polyacrylamide gel electrophoresis (PAGE) and then the 18–30 nt size range of RNA was enriched for purification. The purified small RNA was subjected to sequencing with an Illumina Genome Analyzer (Illumina) according to the manufacturer’s instructions. Briefly, proprietary adapters were ligated to the 5ʹ and 3ʹ termini of the small RNA, and the ligated small RNA were then used as templates for cDNA synthesis, and further converted into 62–75 nt single-stranded cDNA. The cDNA were amplified using illumina’s 3′ adaptor reverse primer and 5′ adaptor forward primers. The purified PCR products were sequenced using Solexa’s proprietary sequencing-by-synthesis method. DNA sequencing was performed with an Illumina Genome Analyzer at the BGI (Beijing Genomics Institute, Shenzhen, China).

### Data processing and mapping sequence reads to the reference genome

All raw datasets produced by deep sequencing from each library (25d MF, 25d SF) were subjected to further analysis. Clean reads were obtained after removal of low quality reads, insert null reads, adaptor null reads, 5′ adaptor contaminants, reads with poly (A) tails and reads shorter than 18 nt. Adapter sequences were then trimmed from both ends of the clean reads. The small RNA with sizes ranging from 18–30 nt were used for further analyses. The clean reads in each library were mapped onto the draft *S.japonicum* genome sequences (ftp://down:lsbi@lifecenter.sgst.cn:2121/subjectData/schistosoma/) and (ftp://ftp.sanger.ac.uk/pub/pathogens/Schistosoma/mansoni/genome/smav5.2.chr.fa) using the Short Oligonucleotide Alignment Program (SOAP). The small RNA that matched with known rRNA, tRNA, small nuclear RNA (snRNA), and small nucleolar RNA (snoRNA) deposited in the Rfam database (ftp://selab.janelia.org/pub/Rfam/) and NCBI GenBank (https://www.ncbi.nlm.nih.gov/GenBank) were excluded. Then, we aligned the predicted miRNA to known miRNA or homologous miRNA in the miRBase (version 21, https://microrna.sanger.ac.uk) to identify conserved miRNA Other small RNA were further analyzed to predict new miRNA by exploring the secondary structure, the Dicer cleavage site and minimum free energy of the unannotated small RNA tags that could be mapped to the genome using MIREAP (https://sourceforge.net/projects/mireap).

### Bioinformatics analysis of *S. japonicum* small RNA

Small RNA tags were aligned to the miRNA precursor/mature miRNA of corresponding species in miRBase20. Detailed information of the alignment, including the structure of known miRNA precursors, and the length and count of tags from the sample, among others, were collected. To make every unique small RNA mapped to only one annotation, we followed the following priority Ruler RNA etc.: rRNA etc. (in which Genbank > Rfam) > known miRNA > repeat > exon > intron3. The total rRNA proportion should be less than 40%. The expression of the miRNA in two samples were visualized by plotting the Log2-ratio figure and the scatter plot. The procedures adopted were as follows: (1) the expression of miRNA in two samples (25d MF and 25d SF) were normalized to get the expression in transcripts per million (TPM) with the normalization formula (normalized expression = actual miRNA count/total count of clean reads × 1 000 000); and (2) the fold-change and P-value were calculated from the normalized expression, and log2-ratio and scatter plots were generated using the fold-change formula (fold change = log2 (25d MF/25d SF)).

### MiRNA quantification by quantitative RT-PCR analysis

Differentially expressed miRNA were examined using qPCR with SYBR green. The first cDNA strand was synthesized from 0.2 μg total RNA using PrimeScript® RT reagent Kit (Perfect Real Time, TaKaRa, Otsu, Japan, code: DRR037A). Stem-loop qRT-PCR was performed to quantify the sex-biased expressed miRNA. Stem-loop RT primers (Table 1) were used to reverse-transcribe mature miRNA to cDNA. The RNA templates for the qPCR were performed on the same samples used for Solexa deep sequencing. The 20 μL reaction RT system contained 2 μL of total RNA (0.2 μg), 0.4 μL (10 μM) of each individual stem-loop RT primer, 0.4 μL ROX Reference Dye II (50 ×), 10 μL SYBR Premix Ex Taq (2 ×), and 6.8 μL Easy Dilution. The RT primers of the miRNA, the forward primers of miRNA and the common reverse primer were designed as shown in Table 1. The cDNA were synthesized by incubation for 30 min at 16 °C, 45 min at 37 °C, and 15 s at 85 °C. The products were amplified using SYBR® Premix ExTaq (TliRNaseH Plus, TaKaRa, code: RR420A) in an ABI Prism 7500 sequence detection system (Applied Biosystems, Waltham, MA, USA) according to the manufacturer’s instruction. Our system contained 2 μL of cDNA from the RT reaction product, 5 μL of 2 × SYBR Premix ExTaq (TaKaRa), and 0.4 μL of 10 μM forward and reverse primers. The NADPH gene was used in the qRT-PCR reaction as an endogenous reference [8]. Relative levels of gene expression were calculated using the 2^ − △△Ct^ method. All assays were performed in triplicate.

**Table 1:** **Primers of reverse transcription and RT-PCR.**

Primer	Primer sequence (5′ → 3′)
NADPH forward	GAGGACCTAACAGCAGAGG
NADPH reverse	TCCGAACGAACTTTGAATTC
Common reverse	GTGCAGGGTCCGAGGT
Let-7 SLP	GTTGGCTCTGGTGCAGGGTCCGAGGTATTCGCACCAGAGCCAACACCACA
Let-7 RP	GTGTTTTG GGAGGTAGTTCGTTG
Mir-1 SLP	GTTGGCTCTGGTGCAGGGTCCGAGGTATTCGCACCAGAGCCAACGACCAT
Mir-1 RP	GTGTTTTGTGGAATGTGGCGAAGT
Mir-10-5p SLP	GTTGGCTCTGGTGCAGGGTCCGAGGTATTCGCACCAGAGCCAACCCAAAC
Mir-10-5p RP	GTGTTTTG AACCCTGTAGACCCGA
Mir-61-5p SLP	GTTGGCTCTGGTGCAGGGTCCGAGGTATTCGCACCAGAGCCAACAAGTGA
Mir-61-5p FP	GTGTTTTGTGACTAGAAAGTGCAC
Mir-124-3p SLP	GTTGGCTCTGGTGCAGGGTCCGAGGTATTCGCACCAGAGCCAACTGACAT
Mir-124-3p RP	GTGTTTTG TAAGGCACGCGGTGA
Mir-3479-3p SLP	GTTGGCTCTGGTGCAGGGTCCGAGGTATTCGCACCAGAGCCAACCAAGGC
Mir-3479-3p RP	GTGTTTTG TATTGCACTTACCTTC

### Prediction and bioinformatics analysis of differentially expressed miRNA target genes

The regulatory effect of differentially expressed miRNA in 25d MF and 25d SF were predicted by analyzing the functions of their predicted target genes and by comparing the proportion of their predicted target genes in differentially expressed genes. In this study, RNA hybrid (https://140.109.42.4/cgi-bin/RNAhybrid/RNAhybrid.cgi) [11] were used to predict the target genes of differentially expressed miRNA. The predicted target genes were filtered with minimum free energy (MFE <  = -20 kcal/mol). These target genes were re-filtered by comparison with the RNA-Seq (Quantification) transcriptome results of SF and MF. The worms were the same sets used in miRNA deep sequencing (Data not published); the genes that were not consistent in the gene expression profile and miRNA target gene profile were excluded. Then those corresponding to target genes were mapped to the terms in the Gene Ontology (GO) database, to identify significantly enriched GO terms in DEG (differentially expressed genes) compared with the genome background. Pathway enrichment analysis was used to identify significantly enriched metabolic pathways or signal transduction pathways in DEG compared with the whole genome background. Detailed pathway information was determined with the KEGG database.

## Results

### Host animals and parasites

**Figure 1:**
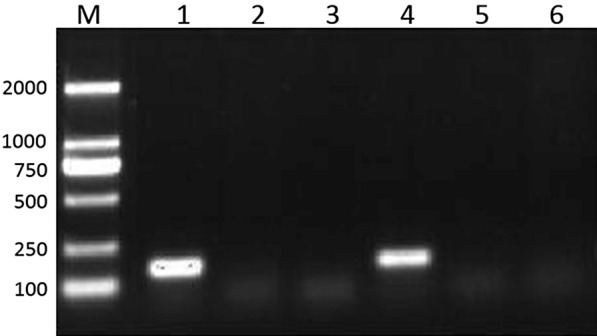
**Identification of the sex of cercariae shed from different snails by PCR.** Lane M, DNA Marker, Lanes 1–6, Cercariae shed from different snails.

Cercariae were collected from individual snails and their sex was identified by PCR. There is a 153 bp specific band from female *S. japonicum* cercariae, and no band from male *S. japonicum* cercariae (Figure 1). For those snails that contain two sex cercariae, the SF female worms were guaranteed by sacrificing the mice that were infected by cercariae from the same snail when the worms were collected.

### Preliminary analysis of raw reads of miRNA in *S. japonicum* 25d MF and 25d SF

**Figure 2:**
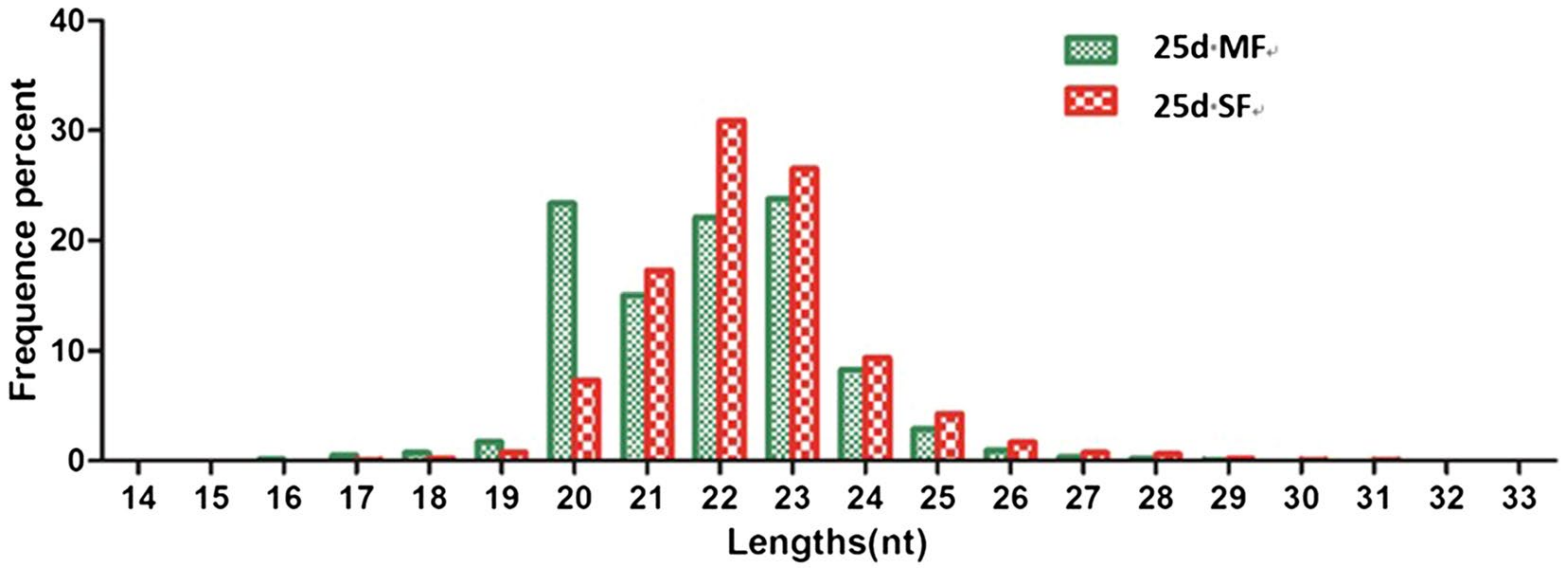
**Length, distribution and abundance of small miRNA tags of 25d MF and 25d SF.**

The cDNA libraries derived from 18–30 nt long RNA isolated from 25d MF and 25d SF were constructed and sequenced using an Illumina (Solexa) DNA sequencer, yielding a total of 9 358 376 and 9 314 275 clean reads corresponding to 97.74% and 97.29% of high-quality sequence for *S. japonicum* 25d MF and 25d SF, respectively (Table 2). The length distribution of the miRNA from the two libraries mainly ranged from 20–24 nt (Figure 2).

**Table 2:** **Proportion of clean reads and contaminant reads that were removed.**

	25d MF		25d SF	
	Amounts	Percentage (%)	Amounts	Percentage (%)
Total reads	9 600 000	–	9 600 000	–
High-quality reads	9 574 851	100	9 574 050	100
3′ adapter null	1879	0.02	1904	0.02
	6190	0.06	8090	0.08
5′adapter contaminants	148 410	1.55	238 250	2.49
Smaller than 18nt	59 894	0.63	11 273	0.12
Poly(A)	102	Less than 0.01	258	Less than 0.01
Clean reads	9 358 376	97.74	9 314 275	97.29

### Differential expression profiles of miRNA in *S. japonicum* 25d MF and 25d SF

All the differentially expressed miRNA profile data for *S. japonicum* 25d MF and 25d SF were normalized to TPM as previously described [11–13]. As shown in Table 3, statistical analysis revealed that 36 *S. japonicum* miRNA were differentially expressed in the 25d SF and 25d MF. 20 miRNA were up-regulated (fold-change (log2 (25d MF/25d SF)) > 1), and 16 miRNA were down-regulated (fold-change (log2 (25d MF/25d SF)) < -1) in 25d MF.

**Table 3:** **Differentially expressed miRNA of 25d SF and 25d MF.**

miR-name	25d SF	25d MF	Fold-change (log2(MF/SF))	Sig-label
sja-miR-3485-5p	0.01	2.0303	7.66554911	**
sja-miR-3491	1.7178	32.9117	4.25996657	**
sja-miR-3501	0.9663	17.6313	4.18952388	**
sja-miR-3497	0.4294	6.9456	4.01570523	**
sja-miR-3506	0.8589	11.113	3.69361435	**
sja-miR-3486-5p	0.5368	5.9839	3.47862949	**
sja-miR-3493	2.362	24.5769	3.37922208	**
sja-bantam	182.5155	1745.3883	3.25745714	**
sja-miR-3490	8.2669	76.8296	3.2162439	**
sja-miR-3504	1.3957	7.9074	2.50221453	**
sja-miR-3498	2.362	9.4033	1.99315818	**
sja-miR-3502	3.3282	13.0364	1.96973149	**
sja-miR-3489	9.4479	33.9803	1.84663299	**
sja-miR-219-3p	0.4294	1.496	1.80071608	*
sja-miR-3494	10.5215	32.5911	1.63113765	**
sja-miR-3500	8.0522	24.4701	1.60356509	**
sja-miR-3507	7.8374	21.7987	1.47579506	**
sja-miR-3495	12.1319	27.8895	1.20091656	**
sja-miR-3505	27.9141	61.9766	1.15072957	**
sja-miR-3488	3.5429	7.3731	1.05734058	**
sja-miR-2c-5p	97.5921	44.2384	− 1.14146516	**
sja-miR-1	227 748.0534	92 404.601	− 1.30140213	**
sja-let-7	10 770.2425	4251.9129	− 1.34086678	**
sja-miR-124-3p	3050.8011	1120.4936	− 1.44505372	**
sja-miR-3482-3p	3.5429	1.2823	− 1.46619692	**
sja-miR-7-5p	12 562.7598	4298.5022	− 1.54724748	**
sja-miR-3503	11.9172	3.9537	− 1.59177	**
sja-miR-36-3p	1457.1182	444.201	− 1.71383337	**
sja-miR-10-5p	2740.3099	826.746	− 1.72882299	**
sja-miR-3479-3p	255.4144	73.9445	− 1.78832512	**
sja-miR-124-5p	4.1871	1.1754	− 1.83279957	**
sja-miR-8-3p	79.8774	20.8369	− 1.93864672	**
sja-miR-36-5p	3.0061	0.6411	− 2.22927169	**
sja-miR-125a	4450.2659	910.8418	− 2.28861913	**
sja-miR-3482-5p	2.1472	0.4274	− 2.32879776	**
sja-miR-133	4.0798	0.748	− 2.44738825	**

Sig-label: Significance label: **p value < 0.01, *0.01 < *p* value < 0.05.

**Figure 3:**
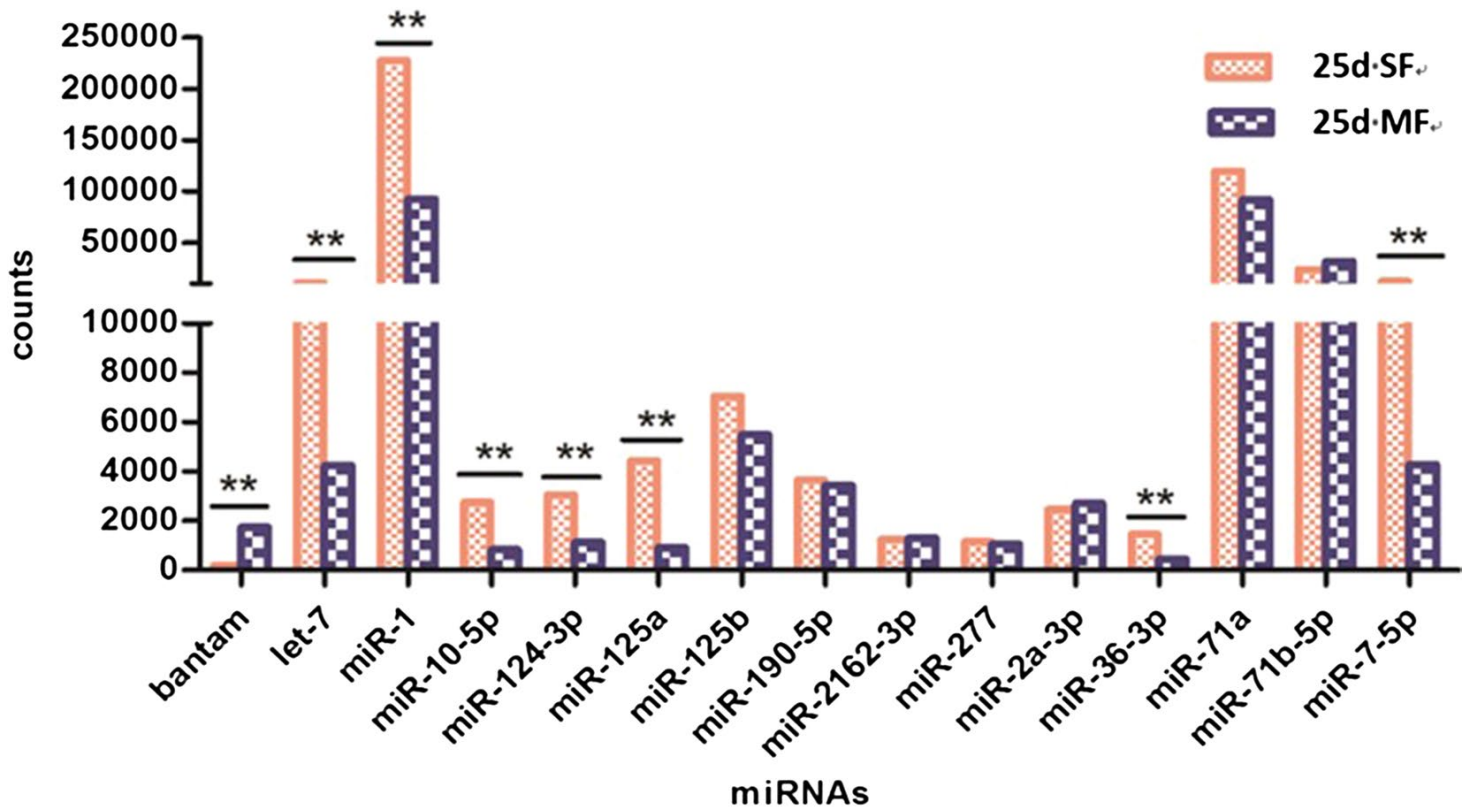
Comparison of the miRNA profiles from 25d SF and 25d MF. **Fold-change (log2) > 1 or fold-change (log2) < -1, and *P* value < 0.01.

Some high-abundance miRNA, such as ban, let-7, miR-1, miR-7-5p and so on, were significantly differentially expressed between 25d MF and 25d SF. Some high-abundance miRNA, however, such as miR-125b, miR-190-5p, miR-2162-3p, miR-277, miR-2a-3p, miR-71a and miR-71b-5p, were not significantly differentially expressed between the two samples (Figure 3). Meanwhile, some low-abundance miRNA, such as miR-3490, miR-3479-3p, miR-3505, miR-2c-5p (Table 3), were significantly differentially expressed too, and there are also some low-abundance miRNA that were not differentially expressed (Data not shown). Some miRNA that were functionally characterized as developmental-related in other organisms were differentially expressed in these two samples, such as ban, let-7, miR-124, miR-8, miR-1, and miR-7.

### Confirmation of differentially expressed miRNA by quantitative RT-PCR analysis

**Figure 4:**
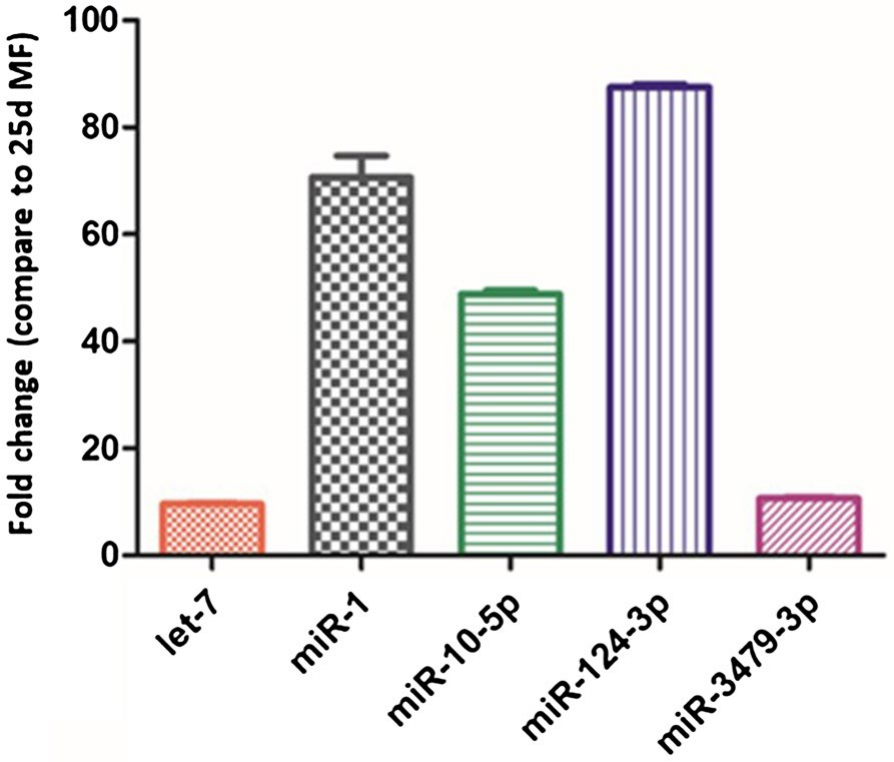
**Result of qPCR confirmation of miRNA expression.**

To confirm the differential expression of miRNA in 25d MF and 25d SF, let-7, miR-1, miR-10-5p, miR-124-3p and miR-3479-3p were selected for quantitative RT-PCR analysis. The results of the RT-PCR showed that let-7, miR-1, miR-10-5p, miR-124-3p and miR-3479-3p were expressed higher in 25d SF than in 25d MF (Figure 4). These results were consistent with the Solexa analysis.

### Predictions and bioinformatics analysis of the predicted target genes for differentially expressed miRNA

**Figure 5:**
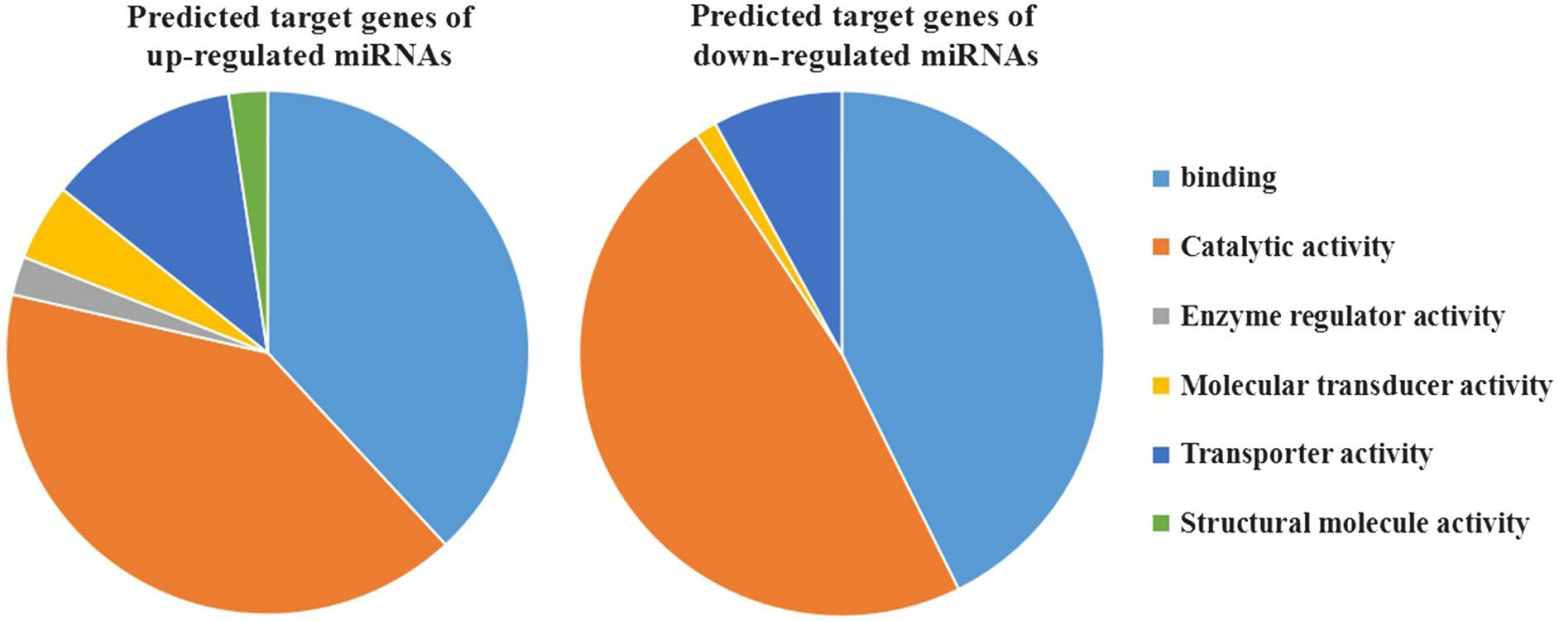
**GO terms of molecular-function in the target genes of up- and down-regulated miRNA.**

To analyze the effect of the differential expression of miRNA on 25d MF and 25d SF, the target genes of the differentially expressed miRNA between the two samples were predicted and analyzed. The target genes of up- or down-regulated miRNA were predicted and analyzed separately. Based on the target gene prediction software, 4728 and 3906 target genes of up- and down-regulated miRNA were acquired respectively. After comparison with the RNA-Seq transcriptome results, there were 166 target genes of up-regulated miRNA and 201 target genes of down-regulated miRNA left respectively. The bioinformatics analysis for target genes of either up- or down-regulated miRNA was applied separately. Not all the target genes can be applied to GO terms, for example, only 41 out of 166 target genes of up-regulated miRNA and 76 out of 201 target genes of down-regulated miRNA were logged in the GO terms of molecular function (Additional file 1). The target genes of up- or down-regulated miRNA were mainly concentrated on the same GO terms (Additional file 2 and Additional file 3). For molecular-function, they were both concentrated on binding, catalytic activity, molecular transducer activity, transporter activity (Figure 5). But the specific target genes of each GO term were different for up- or down-regulated miRNA and there were also target genes of up- and down-regulated miRNA concentrated on different GO terms (Figure 5). For biological process and cellular component GO terms, the same situation was found (Additional file 4 and Additional file 5).

Some target genes of up- or down-regulated miRNA were selected to analyze their functions. The results show that target genes were not only regulated by only one miRNA, such as the target gene Sjc_0005980, but that a target gene can be involved in more than one GO term (Additional file 6 and Additional file 7). This means they participate in multiple important biological processes.

Pathway enrichment analysis also testified to this situation. KEGG results show that some target genes of up- and down-regulated miRNA take part in the same pathway, but the individual target genes in up- and down-regulated miRNA are different. In addition also some target genes of up- or down-regulated miRNA are involved in various pathways, however, only target genes of up-regulated miRNA are involved in these pathways, such as starch and sucrose metabolism, carbohydrate digestion and absorption, TGF-beta signaling pathway, chemokine signaling pathway, and so on. For the pathways, protein processing in the endoplasmic reticulum, N-glycan biosynthesis, ribosome biogenesis in eukaryotes, etc., only target genes of down-regulated miRNA take part (Table 4).

**Table 4:** **Comparison of predicted target genes participating in regulating metabolic processes between up-regulated miRNAs and down-regulated miRNAs in 25d MF.**

Metabolic processes in KEGG	Target genes with pathway annotation (103) of up-regulated miRNAs	Target genes with pathway annotation (147) of down-regulated miRNAs
Regulation of actin cytoskeleton	15 (14.56%)	3 (2.04%)
Focal adhesion	12 (11.65%)	3 (2.04%)
Protein digestion and absorption	7 (6.8%)	1 (0.68%)
Tight junction	7 (6.8%)	4 (2.72%)
Calcium signaling pathway	6 (5.83%)	4 (2.72%)
Phagosome	5 (4.85%)	6 (4.08%)
Mineral absorption	4 (3.88%)	3 (2.04%)
Gastric acid secretion	4 (3.88%)	4 (2.72%)
Pancreatic secretion	4 (3.88%)	3 (2.04%)
Insulin signaling pathway	4 (3.88%)	2 (1.36%)
Ubiquitin mediated proteolysis	3 (2.91%)	2 (1.36%)
Endocytosis	3 (2.91%)	5 (3.4%)
Lysosome	2 (1.94%)	12 (8.16%)
Oocyte meiosis	1 (0.97%)	8 (5.44%)
Long-term potentiation	1 (0.97%)	4 (2.72%)
Salivary secretion	1 (0.97%)	5 (3.4%)
Starch and sucrose metabolism	8 (7.77%)	0 (0%)
Galactose metabolism	6 (5.83%)	0 (0%)
Carbohydrate digestion and absorption	6 (5.83%)	0 (0%)
Axon guidance	4 (3.88%)	0 (0%)
TGF-beta signaling pathway	2 (1.94%)	0 (0%)
Chemokine signaling pathway	2 (1.94%)	0 (0%)
Proximal tubule bicarbonate reclamation	2 (1.94%)	0 (0%)
Osteoclast differentiation	2 (1.94%)	0 (0%)
Fc gamma R-mediated phagocytosis	2 (1.94%)	0 (0%)
Oxidative phosphorylation	2 (1.94%)	0 (0%)
Protein processing in endoplasmic reticulum	0 (0%)	14 (9.52%)
N-Glycan biosynthesis	0 (0%)	7 (4.76%)
Ribosome biogenesis in eukaryotes	0 (0%)	7 (4.76%)
Gap junction	0 (0%)	6 (4.08%)
Dopaminergic synapse	0 (0%)	4 (2.72%)
Spliceosome	0 (0%)	4 (2.72%)
RNA transport	0 (0%)	4 (2.72%)
GnRH signaling pathway	0 (0%)	4 (2.72%)
Phosphatidylinositol signaling system	0 (0%)	4 (2.72%)
DNA replication	0 (0%)	4 (2.72%)
Long-term depression	0 (0%)	4 (2.72%)

## Discussion

The MF worms of *S. japonicum* can develop to sexual maturation and maintain this mature state by pairing with male worms [14]. The SF worms were considerably different in size and structure of inner tissues or systems from those MF worms [15]. Although the long term infected SF can lay very few eggs, these non-viable eggs do not contain developing or live miracidia so there are no soluble miracidial antigens that can induce the host’s immune response, therefore the pathological reaction in infected final host and the schistosomiasis spreading in the environment will be relieved or stopped [16]. During the development of *S. japonicum*, males and females begin to pair at 15 or 16 d post-infection, and females begin to lay eggs from 24 d post-infection [14]. So *S. japonicum* will develop totally mature worms at 25 d post-infection. In this study, the miRNA profiles of 25d MF and 25d SF were compared, some differentially expressed miRNA in the two samples were found, and some clues about the correlation between differentially expressed miRNA and the mature status of schistosome female worms appeared by analyzing the differentially expressed miRNA and their target genes.

The adult female and male worms have obvious gender dimorphism, which can be identified by naked eyes, but the sex of miracidium, sporocyst and cercariae cannot be identified in this way, as they do not have obvious gender dimorphism. One snail can be infected by several miracidia, but in the majority of snails (65.7%), only one miracidium can develop into a mother sporocyst [17]. The proportion of single- or mixed-sex infections of parasites within snail individuals in the field has been reported for *S. japonicum*, most cercariae shed from an individual snail were single-sex [18]. Based on this situation, in this study, the sex of cercariae overflow from an individual snail was confirmed by PCR and infected mice were sacrificed, to ensure that SF worms were recovered.

There are significant morphological differences between normal developed and underdeveloped female *S. japonicum*. In our previous research, the underdeveloped female *S. japonicum* was significantly shorter and thinner than normal developed, and the mature ootid in the indistinct ovary of the underdeveloped female could not be observed [19]. These distinct morphological changes may be associated with miRNA regulation, in this study; there are 20 up-regulated and 16 down-regulated miRNA in 25d MF. The differentially expressed miRNA: miR-1, miR-124, miR-8, miR-7, let-7, and ban, have been functionally characterized as developmental regulators in other organisms [20].

Bantam is a miRNA that is functionally well characterized in *Drosophila*. In *Drosophila*, it is a developmentally regulated miRNA that controls cell proliferation and regulates the proapoptotic gene hid [21]. Bantam was also identified with genes promoting organ growth and systemic body growth by connecting insulin signaling, and ecdysone production [22]. Boulan et al. found that lower ban activity induced growth cessation of late ecdysone-producing cells that led to the cells entry into a metamorphosis stage, whereas ban’s high activity promotes the larvae’s systemic growth in *Drosophila* [22]. In our study, ban was also up-regulated in developed females and down-regulated in underdeveloped females (Table 3). These phenomena are also observed in *Caenorhabditis elegans*, the deletion of ban orthologs in *Caenorhabditis elegans* cause severe growth defects and prevent entry into the dauer state [23].

In this study, two miRNA, miR-7 and miR-1, which have been characterized as tumor suppressors in many kinds of human cancers, were up-regulated in 25d SF. They were found to play a key role in cell proliferation and survival either by being involved in the insulin-like growth factor 1 (IGF1) receptor signaling pathway or epidermal growth factor (EGF) receptor signaling pathway. EGF is an early growth response gene that is induced by mitogenic stimuli, including serum and purified factors such as IGF, EGF, and TGF-beta [24]. Let-7 and miR-124 were also up-regulated in 25d SF in our study. The Let-7 miRNA family has an important function in cell differentiation and cancer [25]. Worringer et al. suggested that let-7 based pathways are an inhibitory influence on the reprogramming process through a regulatory pathway involving prodifferentiation factors, including EGF receptor 1 [26]. MiR-124 also plays a key role in cell differentiation, especially in the nervous system [27]; it was also thought to be a growth suppressor [28]. MiR-8, recognized as a critical mediator for several well characterized developmental regulatory networks such as Wnt, Notch, and TGF-beta in *Drosophila* or mammalian cells [29, 30], is up-regulated in 25d SF too. In addition, the Wnt, Notch, and TGF-beta pathways are shown to be involved in the parasite development, oogenesis and embryogenesis in *schistosoma mansoni* [31–34]. The up-regulation of these differentiation-associated miRNA in 25d SF may be related to the underdevelopmental status of SF. Sexual immaturity may mean more potential for differentiation when a single female pairs with her mate.

The bioinformatics analysis of the predicted target genes for differentially expressed miRNA shows that the target genes distributed in different GO terms, which means these differential functional genes, take part in many important biological processes. The KEGG results show that more target genes in 25d MF are involved in the regulation of actin cytoskeleton, focal adhesion, protein digestion and absorption, tight junction, and calcium signaling pathway metabolic processes whereas more target genes in 25d SF are involved in lysosomes, oocyte meiosis, long-term potentiation and salivary secretion pathways. Only target genes of 25d MF were detected in starch and sucrose metabolism, galactose metabolism, carbohydrate digestion and absorption, axon guidance, TGF-beta signaling pathway, and chemokine signaling pathways whereas only target genes in 25d SF were found in pathways involved in protein processing in the endoplasmic reticulum, N-glycan biosynthesis, ribosome biogenesis eukaryotes, gap junction, dopaminergic synapse, spliceosome, RNA transport, GnRH signaling pathway, and long-term depression. All these are associated with the developmental stages of female worms.

Some receptors in the Wnt, Notch, IGF, EGF, TGF-beta signaling pathways which interact with miRNA have been characterized in both *S. mansoni* and *S. japonicum* [35, 36], these signaling pathways have also been implicated in critical roles in schistosome development by the *S. japonicum* genome study [37], but direct evidence for miRNA regulated signaling pathways remains to be experimentally clarified. This study shows that some miRNA could regulate the sexual development of female worms and that it analyzed target genes. This will shed some clues on the understanding of the development mechanism of schistosome female worms.

## Supplementary information

**Additional file 1. GO terms of molecular-function in the target genes of up- and down-regulated miRNAs.**

**Additional file 2. GO term analysis of the target gene of up-regulated miRNA.**

**Additional file 3. GO term analysis of the target gene of down-regulated miRNA.**

**Additional file 4. The target genes of up-regulated miRNAs GO terms.**

**Additional file 5. The target genes of down-regulated miRNAs GO terms.**

**Additional file 6. Selected predicted target genes of up-regulated miRNAs.**

**Additional file 7. Selected predicted target genes of down-regulated miRNAs.**

## Data Availability

The microRNA sequence data were deposited in the SRA database at NCBI with SRA, Project accession number: PRJNA648823.

## Abbreviations

*S. japonicum*: *Schistosoma japonicum*
RNA: ribonucleic acid
SF: the single-sex infected female worms
DNA: deoxyribonucleic acid
MF: the bisexual infected mature female worms
PCR: polymerase chain reaction
PAGE: polyacrylamide gel electrophoresis
qRT-PCR: quantitative real-time PCR
NADPH: nicotinamide adenine dinucleotide phosphate
DEGs: differentially expressed genes
SOAP: short Oligonucleotide Alignment Program
TPM: transcripts per million
MFE: minimum free energy
GO: gene Ontology
KEGG: Kyoto Encyclopedia of Genes and Genomes
PBS: phosphate-bufered saline
Fig: figure
IGF: insulin-like growth factor
EGF: epidermal growth factor
TGF: transforming growth factor
