# Supplementary material for: Differential expression of microRNA between normally developed and underdeveloped female worms of Schistosoma japonicum
Source: Vet Res. 2020 Sep 25;51:126. doi: 10.1186/s13567-020-00851-4 (PMC7519503; doi:10.1186/s13567-020-00851-4)
Supplement: Supplementary file 1 — Additional file 1. GO terms of molecular-function in the target genes of up- and down-regulated miRNAs. [file 13567_2020_851_MOESM1_ESM.docx]

**Table S1. GO terms of molecular-function in the target genes of up- and down-regulated miRNAs**

| Molecular-function | Predicted target genes of up-regulated miRNAs (41)  Amounts (Percentage（%）) | Predicted target genes of  down-regulated miRNAs (76)  Amounts (Percentage（%） |
| --- | --- | --- |
| binding | Sjc_0056260; Sjc_0030100; Sjc_0091950; Sjc_0005980; Sjc_0017990; Sjc_0131210; Sjc_0001610; Sjc_0054830; Sjc_0063230; Sjc_0027970; Sjc_0043990; Sjc_0121800; Sjc_0001640; Sjc_0095240; Sjc_0081760; Sjc_0036560  16 (39.02%) | Sjc_0035070; Sjc_0065230; Sjc_0032110; Sjc_0035130; Sjc_0082920; Sjc_0025540; Sjc_0112300; Sjc_0015820; Sjc_0025810; Sjc_0023460; Sjc_0086300; Sjc_0115020; Sjc_0069310; Sjc_0036690; Sjc_0077300; Sjc_0018000; Sjc_0022840; Sjc_0039940; Sjc_0000920; Sjc_0022470; Sjc_0000740; Sjc_0082530; Sjc_0070270; Sjc_0052070; Sjc_0075600; Sjc_0004870; Sjc_0046120; Sjc_0019530; Sjc_0042400; Sjc_0005910; Sjc_0091980; Sjc_0002140  32 (42.11%) |
| Catalytic activity | Sjc_0085200; Sjc_0068110; Sjc_0081760; Sjc_0031630; Sjc_0095240; Sjc_0027340; Sjc_0131210; Sjc_0038180; Sjc_0017990; Sjc_0033580; Sjc_0034690; Sjc_0078540; Sjc_0009630; Sjc_0026410; Sjc_0126800; Sjc_0028730; Sjc_0000030  17 (41.46%) | Sjc_0035400; Sjc_0106630; Sjc_0086160; Sjc_0026200; Sjc_0018570; Sjc_0002140; Sjc_0019530; Sjc_0055090; Sjc_0082920; Sjc_0112300; Sjc_0105130; Sjc_0091980; Sjc_0046120; Sjc_0032110; Sjc_0004870; Sjc_0086300; Sjc_0005910; Sjc_0037010; Sjc_0000920; Sjc_0058200; Sjc_0022840; Sjc_0000740; Sjc_0046190; Sjc_0075600; Sjc_0052070; Sjc_0030900; Sjc_0035070; Sjc_0022170; Sjc_0060870; Sjc_0001230; Sjc_0006800; Sjc_0042800; Sjc_0015820; Sjc_0023460; Sjc_0031570; Sjc_0025540  36 (47.37%) |
| Enzyme regulator activity | Sjc_0117060  1 (2.44%) |  |
| Molecular transducer activity | Sjc_0095240; Sjc_0028820  2 (4.88%) | Sjc_0056230  1 (1.32%) |
| Transporter activity | Sjc_0001530; Sjc_0010110; Sjc_0081760; Sjc_0000030; Sjc_0009630  5 (12.20%) | Sjc_0000920; Sjc_0105130; Sjc_0037010;  Sjc_0079140; Sjc_0075410; Sjc_0019480  6 (7.89%) |
| Structural molecule activity |  | Sjc_0069310  1 (1.32%) |
