# Supplementary figures and images for: Differential expression of microRNA between normally developed and underdeveloped female worms of Schistosoma japonicum
Source: Vet Res. 2020 Sep 25;51:126. doi: 10.1186/s13567-020-00851-4 (PMC7519503; doi:10.1186/s13567-020-00851-4)

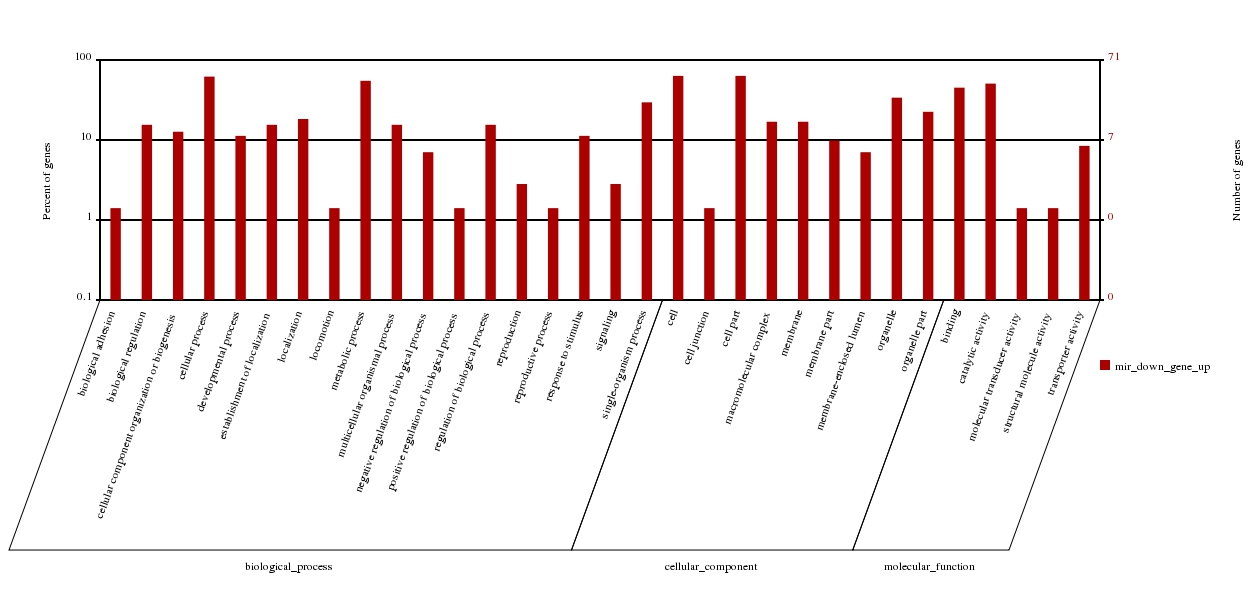

Supplement: Supplementary file 2 — Additional file 2. GO term analysis of the target gene of up-regulated miRNA. [file 13567_2020_851_MOESM2_ESM.jpg]

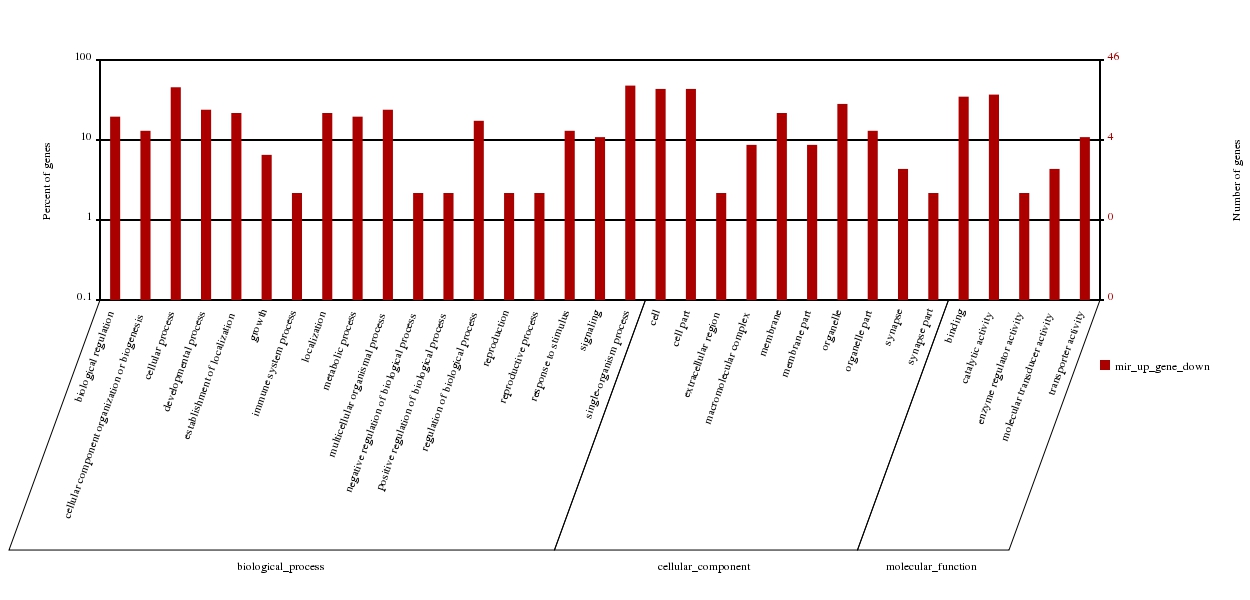

Supplement: Supplementary file 3 — Additional file 3. GO term analysis of the target gene of down-regulated miRNA. [file 13567_2020_851_MOESM3_ESM.jpg]
