# Supplementary material for: Differential expression of microRNA between normally developed and underdeveloped female worms of Schistosoma japonicum
Source: Vet Res. 2020 Sep 25;51:126. doi: 10.1186/s13567-020-00851-4 (PMC7519503; doi:10.1186/s13567-020-00851-4)
Supplement: Supplementary file 6 — Additional file 6. Selected predicted target genes of up-regulated miRNAs. [file 13567_2020_851_MOESM6_ESM.docx]

**Table S4. Selected predicted target genes of up-regulated miRNAs**

| GeneID | 25d SF-RPKM | 25d MF-RPKM | P-value | FDR | GO Component | GO Function | GO Process | Blast nr |
| --- | --- | --- | --- | --- | --- | --- | --- | --- |
| Sjc_0001640\|Sjp_0001640\|SJC_S000003.924123/miR-3505/-1.431396959 | 70.47460602 | 26.12998956 | 1.02523E-05 | 0.000140011 | GO:0005634//nucleus | GO:0003730//mRNA 3'-UTR binding | GO:0060856//establishment of blood-brain barrier; GO:0036093; GO:0007293//germarium-derived egg chamber formation | gi\|360043584\|emb\|CCD81130.1\|/0/elav (embryonic lethal, abnormal vision,drosophila)-like protein [Schistosoma mansoni] |
| Sjc_0005980\|Sjp_0005980\|SJC_S000018.784920/miR-3485-5p, miR-3494, miR-3500, miR-3501, miR-3505/-1.251098369 | 151.1217086 | 63.49049577 | 4.7846E-21 | 3.18147E-19 | - | - | - | gi\|256070624\|ref\|XP_002571643.1\|/0/sno and ski oncogene family [Schistosoma mansoni] |
| Sjc_0017680\|Sjp_0017680\|SJC_S000085.514615/sja-miR-3495/-2.639983581 | 125.6710986 | 20.16142227 | 1.69992E-14 | 7.30376E-13 | GO:0005856//cytoskeleton | - | GO:0007507//heart development;GO:0009653//anatomical structure morphogenesis | gi\|226481587\|emb\|CAX73691.1\|/0/putative LIM domain binding 3 [Schistosoma japonicum] |
| Sjc_0027970\|Sjp_0027970\|SJC_S000172.207150/sja-miR-3502/-1.345961483 | 61.22336666 | 24.08476585 | 3.8609E-07 | 7.01303E-06 | - | - | - | gi\|256087884\|ref\|XP_002580092.1\|/1.41721e-76/axis inhibition protein axin [Schistosoma mansoni] |
| Sjc_0036560\|Sjp_0036560\|SJC_S000258.345983/sja-miR-3500/-3.394871083 | 121.7356089 | 11.5733638 | 3.49242E-24 | 2.67218E-22 | - | GO:0003676//nucleic acid binding | - | gi\|256073766\|ref\|XP_002573199.1\|/7.32581e-179/rna-binding protein musashi-related [Schistosoma mansoni] |
| Sjc_0043990\|Sjp_0043990\|SJC_S000363.385960/sja-miR-3504/-1.329282741 | 160.6233729 | 63.92264889 | 8.94806E-10 | 2.31386E-08 | GO:0005737//cytoplasm | GO:0005536//glucose binding; GO:0003983//UTP:glucose-1-phosphate uridylyltransferase activity; GO:0032557//pyrimidine ribonucleotide binding | GO:0019255//glucose 1-phosphate metabolic process;GO:0006011//UDP-glucose metabolic process | gi\|256074471\|ref\|XP_002573548.1\|/0/utp-glucose-1-phosphate uridylyltransferase 2 (udp-glucose pyrophosphorylase 2) [Schistosoma mansoni] |
| Sjc_0117060\|Sjp_0117060\|SJC_S009046.212/sja-miR-3493/-1.76683986 | 84.6962126 | 24.88812588 | 3.5972E-11 | 1.11934E-09 | - | - | - | gi\|353231019\|emb\|CCD77437.1\|/0/putative neurofibromin [Schistosoma mansoni] |
| Sjc_0098850\|Sjp_0098850\|SJC_S003598.2769/sja-miR-3490/-2.468871665 | 236.9853043 | 42.80721651 | 4.71002E-15 | 2.16525E-13 | - | - | - | gi\|256087458\|ref\|XP_002579886.1\|/5.62335e-113/Collagen alpha-1(V) chain precursor [Schistosoma mansoni] |
| Sjc_0063230\|Sjp_0063230\|SJC_S000754.109/sja-miR-3501/-1.620874758 | 156.7057296 | 50.95102843 | 1.03027E-05 | 0.000140527 | GO:0031430//M band | GO:0046872//metal ion binding; GO:0019904//protein domain specific binding | GO:0071688//striated muscle myosin thick filament assembly | gi\|226479208\|emb\|CAX73099.1\|/9.45551e-128/Four and a half LIM domains protein 2 [Schistosoma japonicum] |
| Sjc_0074210\|Sjp_0074210\|SJC_S001151.1694/sja-miR-219-3p/-3.546874177 | 51.19470052 | 4.380356816 | 1.38209E-05 | 0.00018424 | GO:0031594//neuromuscular junction; GO:0036062; GO:0042734//presynaptic membrane; GO:0043195//terminal button; GO:0005886//plasma membrane | GO:0016787//hydrolase activity; GO:0008092//cytoskeletal protein binding; GO:0005200//structural constituent of cytoskeleton | GO:0000226//microtubule cytoskeleton organization; GO:0007605//sensory perception of sound; GO:0007016//cytoskeletal anchoring at plasma membrane; GO:1900074;GO:0048675//axon extension; GO:0007528//neuromuscular junction development; GO:0070050//neuron homeostasis | gi\|360044067\|emb\|CCD81614.1\|/1.1192e-87/putative ankyrin 2,3/unc44 [Schistosoma mansoni] |
| Sjc_0121800\|Sjp_0121800\|SJC_S014309.1911/sja-miR-3485-5p/-1.289076419 | 76.83197732 | 31.44063736 | 1.25259E-05 | 0.000168383 | GO:0031410//cytoplasmic vesicle; GO:0043234//protein complex | GO:0005515//protein binding; GO:0016874//ligase activity | GO:0035120//post-embryonic appendage morphogenesis; GO:0035114;GO:0030097//hemopoiesis; GO:0021536//diencephalon development; GO:0048259//regulation of receptor-mediated endocytosis; GO:0030902//hindbrain development;GO:0021514//ventral spinal cord interneuron differentiation; GO:0043009//chordate embryonic development; GO:0050767//regulation of neurogenesis; GO:0003002//regionalization; GO:0048839//inner ear development; GO:0001654//eye development; GO:0016567//protein ubiquitination; GO:0048522; GO:0010001//glial cell differentiation; GO:0048514//blood vessel morphogenesis; GO:0007560//imaginal disc morphogenesis; GO:0048646//anatomical structure formation involved in morphogenesis; GO:0048665//neuron fate specification; GO:0002064//epithelial cell development; GO:0008593//regulation of Notch signaling pathway | gi\|256087793\|ref\|XP_002580048.1\|/0/mind bomb [Schistosoma mansoni] |
