# Supplementary material for: Differential expression of microRNA between normally developed and underdeveloped female worms of Schistosoma japonicum
Source: Vet Res. 2020 Sep 25;51:126. doi: 10.1186/s13567-020-00851-4 (PMC7519503; doi:10.1186/s13567-020-00851-4)
Supplement: Supplementary file 7 — Additional file 7. Selected predicted target genes of down-regulated miRNAs. [file 13567_2020_851_MOESM7_ESM.docx]

**Table S5. Selected predicted target genes of down-regulated miRNAs**

| GeneID/miRNA/log2 Ratio(25MSIF/25SSIF) | 25d SF-RPKM | 25d MF-RPKM | P-value | FDR | GO Component | GO Function | GO Process | Blast nr |
| --- | --- | --- | --- | --- | --- | --- | --- | --- |
| Sjc_0000920\|Sjp_0000920\|SJC_S000001.1296743/sja-miR-3503/1.397984269 | 12.52912425 | 33.01839165 | 7.91154E-06 | 0.000110475 | GO:0005886//plasma membrane; GO:0044425//membrane part; GO:0005794//Golgi apparatus | GO:0003824//catalytic activity; GO:0046872//metal ion binding | GO:0043085//positive regulation of catalytic activity; GO:0044238//primary metabolic process; GO:0016043//cellular component organization; GO:0048513//organ development; GO:0030182//neuron differentiation; GO:0006584//catecholamine metabolic process; GO:0050896//response to stimulus; GO:0006825//copper ion transport; GO:0055085//transmembrane transport | gi\|256078528\|ref\|XP_002575547.1\|/0/copper ABC transporter ATPase [Schistosoma mansoni] |
| Sjc_0002140\|Sjp_0002140\|SJC_S000004.940221/sja-miR-36-3p/1.687591077 | 21.41644972 | 68.98632539 | 3.1151E-06 | 4.73453E-05 | GO:0005739//mitochondrion | GO:0030170//pyridoxal phosphate binding; GO:0004372//glycine hydroxymethyltransferase activity | GO:0009792//embryo development ending in birth or egg hatching; GO:0009069//serine family amino acid metabolic process | gi\|226481453\|emb\|CAX73624.1\|/0/serine hydroxymethyltransferase 1 [Schistosoma japonicum] |
| Sjc_0019530\|Sjp_0019530\|SJC_S000102.133869/sja-miR-1/2.130534573 | 39.14834895 | 171.4226735 | 1.90687E-10 | 5.31214E-09 | GO:0043202//lysosomal lumen | GO:0046982//protein heterodimerization activity; GO:0042803//protein homodimerization activity | GO:0005975//carbohydrate metabolic process; GO:0030207//chondroitin sulfate catabolic process; GO:0044707; GO:0030214//hyaluronan catabolic process; GO:0006687//glycosphingolipid metabolic process; GO:0048856//anatomical structure development; GO:0042340//keratan sulfate catabolic process; GO:0065007//biological regulation; GO:0006644//phospholipid metabolic process | gi\|56757485\|gb\|AAW26910.1\|/5.44078e-145/SJCHGC06873 protein [Schistosoma japonicum] |
| Sjc_0022470\|Sjp_0022470\|SJC_S000124.257450/sja-miR-125a,sja-miR-1,sja-miR-3482-3p/1.178349564 | 134.9466617 | 305.4082114 | 6.08308E-36 | 6.79541E-34 | - | - | GO:0044699; GO:0009987//cellular process | gi\|353229203\|emb\|CCD75374.1\|/0/putative scythe/bat3 [Schistosoma mansoni] |
| Sjc_0030900\|Sjp_0030900\|SJC_S000197.495344/sja-let-7,sja-miR-7-5p,sja-miR-124-3p/2.497519943 | 5.567534589 | 31.44063736 | 2.12522E-05 | 0.000269782 | - | GO:0016772//transferase activity, transferring phosphorus-containing groups | GO:0006796//phosphate metabolic process | gi\|353233149\|emb\|CCD80504.1\|/0/ethanolamine-phosphate cytidylyltransferase [Schistosoma mansoni] |
| Sjc_0035070\|Sjp_0035070\|SJC_S000245.225039/sja-miR-36-3p,sja-miR-3503/1.263801104 | 36.68010725 | 88.07905322 | 1.08008E-05 | 0.000146426 | GO:0005634//nucleus | GO:0004674//protein serine/threonine kinase activity; GO:0031625//ubiquitin protein ligase binding; GO:0000287//magnesium ion binding; GO:0008134//transcription factor binding; GO:0005524//ATP binding | GO:0009952//anterior/posterior pattern formation; GO:0006351//transcription, DNA-dependent; GO:0030901//midbrain development; GO:0018107//peptidyl-threonine phosphorylation; GO:0007179//transforming growth factor beta receptor signaling pathway; GO:0031398//positive regulation of protein ubiquitination; GO:0009952//anterior/posterior pattern formation; GO:0001707//mesoderm formation; GO:0033136//serine phosphorylation of STAT3 protein; GO:0030177//positive regulation of Wnt receptor signaling pathway | gi\|56753009\|gb\|AAW24716.1\|/0/SJCHGC09514 protein [Schistosoma japonicum] |
| Sjc_0029900\|Sjp_0029900\|SJC_S000191.483037/sja-miR-3482-5p/1.283603304 | 29.47539776 | 71.75675179 | 1.19099E-13 | 4.71791E-12 | - | - | GO:0034641//cellular nitrogen compound metabolic process; GO:0044238//primary metabolic process; GO:0006725//cellular aromatic compound metabolic process; GO:0046483//heterocycle metabolic process;GO:1901360 | gi\|256075521\|ref\|XP_002574067.1\|/0/hypothetical protein [Schistosoma mansoni] |
| Sjc_0023460\|Sjp_0023460\|SJC_S000133.447692/sja-miR-36-5p/1.982649338 | 53.75443555 | 212.4473056 | 1.29806E-10 | 3.68036E-09 | GO:0005829//cytosol; GO:0005694//chromosome | GO:0004422//hypoxanthine phosphoribosyltransferase activity; GO:0000287//magnesium ion binding; GO:0052657//guanine phosphoribosyltransferase activity; GO:0002060//purine base binding; GO:0042803//protein homodimerization activity | GO:0046038//GMP catabolic process; GO:0006168//adenine salvage; GO:0045964//positive regulation of dopamine metabolic process; GO:0051289//protein homotetramerization; GO:0007417//central nervous system development; GO:0006178//guanine salvage; GO:0032263//GMP salvage; GO:0007610//behavior; GO:0032264//IMP salvage; GO:0043103//hypoxanthine salvage; GO:0006166//purine ribonucleoside salvage; GO:0030182//neuron differentiation | gi\|56682906\|gb\|AAW21808.1\|/1.06905e-130/hypoxanthine-guanine phosphoribosyltranferase [Schistosoma japonicum] |
| Sjc_0030900\|Sjp_0030900\|SJC_S000197.495344/sja-let-7,sja-miR-7-5p,sja-miR-124-3p/2.497519943 | 5.567534589 | 31.44063736 | 2.12522E-05 | 0.000269782 | - | GO:0016772//transferase activity, transferring phosphorus-containing groups | GO:0006796//phosphate metabolic process | gi\|353233149\|emb\|CCD80504.1\|/0/ethanolamine-phosphate cytidylyltransferase [Schistosoma mansoni] |
| Sjc_0018000\|Sjp_0018000\|SJC_S000087.375209/sja-miR-36-3p/1.825679991 | 16.68814264 | 59.15513407 | 2.84812E-06 | 4.36438E-05 | GO:0005925//focal adhesion; GO:0032587//ruffle membrane | GO:0019904//protein domain specific binding | GO:0007492//endoderm development;GO:0032092//positive regulation of protein binding; GO:0070201//regulation of establishment of protein localization; GO:0007398//ectoderm development; GO:0001839//neural plate morphogenesis;GO:0032091//negative regulation of protein binding; GO:0048617//embryonic foregut morphogenesis; GO:0003383//apical constriction; GO:0071560//cellular response to transforming growth factor beta stimulus; GO:0009826//unidimensional cell growth; GO:0048339//paraxial mesoderm development; GO:0022408//negative regulation of cell-cell adhesion; GO:0048319//axial mesoderm morphogenesis; GO:0070986//left/right axis specification; GO:0010608//posttranscriptional regulation of gene expression; GO:0006931//substrate-dependent cell migration, cell attachment to substrate; GO:0051894//positive regulation of focal adhesion assembly; GO:0032525//somite rostral/caudal axis specification;GO:0010718//positive regulation of epithelial to mesenchymal transition;GO:0007509//mesoderm migration involved in gastrulation;GO:0010634//positive regulation of epithelial cell migration;GO:0001701//in utero embryonic development;GO:0031032//actomyosin structure organization | gi\|360043278\|emb\|CCD78691.1\|/0/putative 4.1 G protein [Schistosoma mansoni] |
